# Association between *Porphyromonas Gingivalis* and systemic diseases: Focus on T cells-mediated adaptive immunity

**DOI:** 10.3389/fcimb.2022.1026457

**Published:** 2022-11-17

**Authors:** Cheng Li, Ran Yu, Yumei Ding

**Affiliations:** ^1^ Department of Stomatology, Union Hospital, Tongji Medical College, Huazhong University of Science and Technology, Wuhan, China; ^2^ School of Stomatology, Tongji Medical College, Huazhong University of Science and Technology, Wuhan, China; ^3^ Hubei Province Key Laboratory of Oral and Maxillofacial Development and Regeneration, Wuhan, China

**Keywords:** Porphyromonas gingivalis, periodontal diseases, adaptive immune response, systemic diseases, atherosclerosis, adverse pregnancy outcomes, inflammatory bowel disease, rheumatoid arthritis

## Abstract

The association between periodontal disease and systemic disease has become a research hotspot. *Porphyromonas gingivalis* (*P. gingivalis*), a crucial periodontal pathogen, affects the development of systemic diseases. The pathogenicity of *P. gingivalis* is largely linked to interference with the host’s immunity. This review aims to discover the role of *P. gingivalis* in the modulation of the host’s adaptive immune system through a large number of virulence factors and the manipulation of cellular immunological responses (mainly mediated by T cells). These factors may affect the cause of large numbers of systemic diseases, such as atherosclerosis, hypertension, adverse pregnancy outcomes, inflammatory bowel disease, diabetes mellitus, non-alcoholic fatty liver disease, rheumatoid arthritis, and Alzheimer’s disease. The point of view of adaptive immunity may provide a new idea for treating periodontitis and related systemic diseases.

## 1 Introduction

Periodontal diseases include chronic inflammatory diseases such as gingivitis and periodontitis (PD). With 11% of the world’s population suffering from severe periodontitis, periodontitis is one of the most common periodontal disorders (Hajishengallis et al., 2012). The clinical symptoms and effects of periodontitis include red and swollen gums, bleeding gums, alveolar bone resorption, and even teeth loss, which affect the patient’s ability to chew. Furthermore, there is growing evidence of a strong link between systemic diseases and periodontitis, including atherosclerosis, adverse pregnancy outcomes, inflammatory bowel disease, diabetes mellitus, rheumatoid arthritis, and others.

Periodontal disease is characterized by the immune-mediated destruction of the osseous support surrounding the dentition in the oral mucosa (Darveau, 2010). Periodontitis is an infectious disease resulting from the invasion of periodontal pathogens that activate innate and adaptive immune responses. As periodontitis persists, many immune cells participate in the inflammatory process. While these immune cells deter bacterial invasion, they also secrete various cytokines that cause the periodontal tissues to deteriorate. In this case, a necessary trigger for the initiation and persistence of the inflammatory response is the colonization of the dentition by a microbial biofilm rich in gram-negative bacteria. One of them is *Porphyromonas gingivalis*, an obligate anaerobic bacterium, which is also known as *P. gingivalis* (pg). *P. gingivalis* is believed to be the primary pathogen of periodontal disease because of its capacity to modify the normal oral microbiome to enhance its virulence, which significantly accelerates the process of bone loss (Honda, 2011; Kassebaum et al., 2014). *P. gingivalis* has been closely linked to the emergence of remote inflammatory responses associated with chronic diseases and autoimmune disorders (Pussinen et al., 2007; Martinez-Martinez et al., 2009) in addition to periodontal disease and its consequences (Hajishengallis, 2009). Additionally, *P. gingivalis* is considered a master at subverting the immune system, exploiting several types of sabotage techniques to escape and weaken the immune system (Krauss et al., 2010b). Although having a variety of virulence factors, such as gingipains, lipopolysaccharide, and fimbriae, may seem significant, *P. gingivalis’* pathogenicity is mainly determined by its capacity to undermine the host’s immune system’s defense (Hajishengallis, 2011). Through mechanisms that allow pathogen persistence inside the local inflammatory environment of periodontitis, *P. gingivalis* interferes with the host’s immune system response, leading to pathology or complications at systemic sites. *P. gingivalis* and its virulence factors have been discovered in all parts of the body, including atherosclerotic plaque, placenta, intestine, and joints. Further research has revealed that *P. gingivalis* is a unique bacterium that is capable of infecting myeloid dendritic cells and reprogramming them to cause an immunosuppressive response (Zeituni et al., 2009; Zeituni et al., 2010). There is multiple evidence proving that distant systemic effects are possibly influenced by local microbial dysbiosis and the ensuing immune response within periodontitis (Chapple et al., 2013; Tonetti et al., 2013). Reports have indicated that patients suffering from periodontal diseases may have systemic exposure to *P. gingivalis*, as evidenced by the discovery of *P. gingivalis*-specific T-cells within the peripheral blood (Gemmell et al., 1999; Nakajima et al., 1999). The report also described that in patients suffering from periodontitis and experimental models, the T and B cells were activated in the gingival tissues. These outcomes of the adaptive immune response are antigen-specific to several *P. gingivalis* strains are communicated locally and systemically (Ebersole et al., 2016). A growing number of studies have also suggested that *P. gingivalis* can influence the development of periodontitis-related systemic disorders by affecting the adaptive immune response and inducing inflammation and innate immune responses (**Table 1**).

**Table 1:** *P. gingivalis*-induced adaptive immune responses (mainly mediated by T cells) in various systemic diseases.

Disease	Adaptive immune responses	Reference
Atherosclerosis	T-cell immune response specific to *P. gingivalis* Hsp60(GroEL) may be involved in the immunopathologic process	(Choi et al., 2002; Yamazaki et al., 2004)
	Th17/Treg Cell imbalance (a decrease of Treg cell population and an increase of Th17 cell population)	(Kol et al., 1998; Cai et al., 2014; Yang et al., 2014)
	The dysregulation of Treg cells and reduction of TGF-β1	(Yang et al., 2014)
	Reduction of IL-2	(Khalaf and Bengtssown, 2012)
	Controlling the progression of atherosclerosis through the related vaccination	(Choi et al., 2004; Calder et al., 2006; Koizumi et al., 2008; Choi et al., 2011; Fukasawa et al., 2012; Hagiwara et al., 2014)
Hypertension	Causing Th1 immune response	(Czesnikiewicz-Guzik et al., 2019)
Adverse pregnancy outcomes	Th1/Th2 cytokine Increase in Ratio	(Raghupathy, 2001; Liang et al., 2006)
	Th17/Treg Cell Imbalance	(Pongcharoen et al., 2006; Keeler et al., 2009; Monteiro et al., 2009; Cheng et al., 2014; Figueiredo and Schumacher, 2016)
	Increased production of IL-17	(Dechend et al., 2000; Pongcharoen et al., 2006; Dhillion et al., 2012; Park et al., 2012b; Ozkan et al., 2015)
Inflammatory Bowel Disease	Th17/Treg Cell Imbalance	(Jia et al., 2020; Zhao et al., 2021)
Diabetes Mellitus	Specific antibodies against *P. gingivalis* protect from the impaired glucose metabolism	(Blasco-Baque et al., 2017)
Non-alcoholic fatty liver disease	Th17/Treg Cell Imbalance	(Hatasa et al., 2021; Yao et al., 2022)
Rheumatoid Arthritis	*P. gingivalis* PAD (PPAD) initiate the ACPA response	(McGraw et al., 1999a; Rosenstein et al., 2004; Wegner et al., 2010; Maresz et al., 2013; Gully et al., 2014)
	Stimulates the production of pro-inflammatory mediators and induces Th17 response	(Leipe et al., 2010; Trombone et al., 2010; McInnes and Schett, 2011; van Hamburg et al., 2011; de Aquino et al., 2014; Gonzales et al., 2014)
	The activation of RANK-L pathway which contribute to osteoclastogenesis	(de Pablo et al., 2009; Lu et al., 2018; Narayan et al., 2018)
Alzheimer’s disease	An increasing of Aβ deposition	(Perry et al., 2007; Poole et al., 2013; Nie et al., 2019)
	Increase of the BBB permeability causing the influx of peripheral immune cells and pathogens	(Sadrameli et al., 2020)
	Inhibiting IFN-γ and T cell response	(Poole et al., 2013)

Previous review articles (Hajishengallis, 2015; Olsen et al., 2016; Bui et al., 2019) have discussed the association of inflammation and the innate immune response with periodontal pathogens and systemic disease. However, there has been no comprehensive analysis of the impact of adaptive immunity on the relationship between *P. gingivalis* and periodontitis-related conditions. This review elaborates on how *P. gingivalis* modifies the host’s adaptive immune system by controlling cellular immunological responses, particularly T cells, in relevant systemic diseases. It also offers new ideas for future research and developing treatments for these systemic diseases using adoptive immunotherapy as a treatment strategy.

## 2 *P.gingivalis*-induced immune response

As previously mentioned, *P. gingivalis* is considered a major pathogen in periodontitis, which means that after initially colonizing the host, it can induce dysbiosis in the oral microbiota (Hajishengallis et al., 2012). *P. gingivalis* expresses various virulence factors, such as lipopolysaccharide (LPS), fimbriae, ceramide, nucleoside diphosphate kinase (NDK), capsule, gingipains, and outer membrane vesicles (OMVs) (How et al., 2016), which can trigger host immune responses (summarized in **Table 2**). This periodontopathogen is regarded as an expert at tricking the immune system, and it uses a variety of sabotage strategies to avoid detection by the host immune system or to weaken or trick it (Krauss et al., 2010b). Here, we briefly reviewed how *P. gingivalis* subvert adaptive immune response.

**Table 2:** Immune responses to virulence factors of *P. gingivalis*.

Virulence factors	Immune responses	Reference
Lipopolysaccharide (LPS)	(i) Stimulates bone resorption through the expression of IL-1α and IL-6 via binding to CD14.	(Miyata et al., 1997)
	(ii) The heterogeneity in the lipid A structures of LPS results in opposing host immune responses.	(Reife et al., 2006; Rangarajan et al., 2008)
	(iii) Binds to Toll-like receptors on monocytes, and gingival fibroblasts to stimulate pro-inflammatory cytokines.	(Wang and Ohura, 2002; Diya et al., 2008; Hamedi et al., 2009)
	(iv) Stimulates dendritic cells to induce a cytokine response biased towards an inflammatory Th1 effector response.	(Jotwani and Cutler, 2004)
	(v) Induces hPDLCs to produce IL-17 and IL-23, promoting the environment for Th17 development.	(Park et al., 2012b)
Gingipains	(i) Activates host MMPs; disintegrates cell-cell components, complement system proteins, cytokines, immunoglobulins, integrins, and collagen; and changes cell signaling and cellular function.	(Curtis et al., 2001; Guo et al., 2010)
	(ii) Cleaves T-cell receptors, such as CD2, CD4, and CD8, and affects the cell-mediated immune response. CD14, an endotoxin receptor, is clipped, causing an increase in LPS hypersensitivity.	(Sugawara et al., 2000; Kitamura et al., 2002; Yun et al., 2005)
	(iii) Pro-inflammatory cytokines are released when the expression of protease-activated receptors on neutrophils, gingival epithelial cells, gingival fibroblasts, and T cells are stimulated. This results in bleeding at the periodontal site, an influx of polymorphonuclear leukocytes, and the degradation of fibrinogen to LPS.	(Baba et al., 2001)
	(iv) Degrade and inactivate Th2 anti-inflammatory cytokines such as IL-4 and IL-5.	(Tam et al., 2009)
Capsule (CPS or K-antigen)	(i) The release of chemokines by macrophages and cytokines by dendritic cells is variably stimulated by different serotypes of CPS.	(d'Empaire et al., 2006)
	(ii) More resistance to polymorphonuclear leukocyte phagocytosis and a different ability to attach to the gingival epithelium can both be produced by P. gingivalis strains that have been encapsulated.	(Sundqvist et al., 1991)
Fimbriae	(i) Invasion and colonization potential are acted upon by Type I fimbriae. Type II fimbriae demonstrate greater pro-inflammatory efficacy.	(Amano et al., 2004; Hajishengallis et al., 2008)
	(ii) FimA is involved in the proliferation of autologous CD4+ T cells as well as the secretion of high levels of interferon-γ and lower levels of TNF-α, IL-10, and IL-12, which is consistent with a Th1 effector response.	(Jotwani and Cutler, 2004)
	(iii) Activates cellular receptors such as Toll-like receptors to induce an innate immune response.	(Davey et al., 2008)
Outer membrane vesicles (OMVs)	(i) Purified OMVs of P. gingivalis activate proinflammatory cytokines, promote inflammasome signaling, and cause macrophage pyroptosis.	(Fleetwood et al., 2017)
Phosphoethanolamine dihydroceramide (PEDHC) and phosphoglycerol dihydroceramide (PGDHC)	(i) Promotes IL-1β-mediated release of PGE2 in primary cultures of gingival fibroblasts; induces apoptosis in chondrocytes and gingival fibroblasts; promotes osteoclastogenesis mediated by receptor activator of nuclear factor kappa-B ligand (RANKL) via interaction with Myh9 (nonmuscle myosin II-A) independently of Toll-like receptor 2/4 (TLR2/4)	(Kanzaki et al., 2017)
Serine phosphatase	(i) Involved in neutrophil subversion by dephosphorylating the serine S536 of the p65 subunit of NF-κB and preventing translocation of NF-κB to the nucleus, hence inhibiting IL-8 production.	(Bainbridge et al., 2010; Takeuchi et al., 2013)
Porphyromonas gingivalis peptidylarginine deiminase (PPAD)	(i) Promotes the expression of IL-36γ, IL-8, IL-1β, CCL20, and CXCL8, all of which are categorized as effective immune modulators belonging to the IL-1 system.	(Aliko et al., 2018)
	(ii) Produces ammonia during deimination of arginine to citrulline, which promotes periodontal infection by inhibiting neutrophil function.	(Niederman et al., 1990; Gabarrini et al., 2018)

### 2.1 T cells

Ivanyi and Lehner were the first to discover that oral bacterium encouraged lymphocytes mobilization in patients with mild periodontal disease, whereas this activation was reduced in individuals with severe periodontitis (Ivanyi and Lehner, 1970). Recent studies have demonstrated that the presence of many T cell subsets contributes to the complex roles of T cell immunity in periodontitis. During the development of periodontitis, various subsets of CD4+ T cells support or inhibit the host’s immunological responses (Jin et al., 2014; Wang et al., 2015). In a study by Baker et al., it was shown that rats lacking MHC-II-restricted CD4+ T cells, but not MHC-I-restricted CD8+ T cells, were sensitive to the resorption of oral alveolar bone brought on by *P. gingivalis* infection (Baker et al., 1999). This finding suggests that CD4+ T cells are involved in bone demineralization. CD4+ T cells are divided into distinct functional lineages such as Th1, Th2, Th17, Treg, and some other subsets (Vernal et al., 2014).

#### 2.1.1 Th1/Th2 cells

Th1 and Th2 cells have diverse responses to various stimuli and lead to various results in inflammatory and infectious disorders. It is still controversial that which specific Th subsets are selectively activated during periodontitis. On the one hand, it has been shown that *Porphyromonas gingivalis* and its virulence factors promote Th1 differentiation (Gemmell et al., 1999; Jotwani and Cutler, 2004). On the other hand, *Porphyromonas gingivalis* and its virulence factors have also been reported to induce a Th2 response (Jotwani et al., 2003; Gaddis et al., 2013). It’s interesting to notice that in older animals, a reducing in Th1/Th2 cytokines such IFN-γ, IL-4, IL-12p40, or IL-10 might lead to a more significant loss of alveolar bone (Alayan et al., 2007). Given that IFN-γ and IL-12 are crucial for bacterial cleaning and that IL-4 and IL-10 are well known for their anti-inflammatory properties, the incongruent reports may be explicable by the fact that a delicate balance between pathogens and various host immune elements is necessary to preserve periodontal health.

#### 2.1.2 Th17 cells

Th17 cells have a critical role in both increasing inflammation and defending against extracellular infections and fungi (O'Connor et al., 2010). Early studies have found that patients with periodontitis had the Th17-specific cytokine IL-17 and other relevant cytokines identified in their gingival tissues (Lester et al., 2007). Increased Th17 cell infiltration in periodontal lesions further demonstrated the link between Th17 and periodontitis (Adibrad et al., 2012). Periodontal lesions were encouraged to produce Th17 by *P. gingivalis*, particularly the strain W83 (Moutsopoulos et al., 2012). Different K-serotypes of *P. gingivalis* strains primed dendritic cells, with strains W83 of serotype K1 and HG184 of K2 activating a Th1/Th17 pattern of immune response while strains K3, K4, and K5 stimulated a Th2 response (Vernal et al., 2014). In addition to releasing the pro-inflammatory cytokine IL-17, Th17 cells also affect bone loss by increasing the production of RANKL, which is necessary for osteoclastogenesis (Stadhouders et al., 2018).

#### 2.1.3 Treg cells

Regulatory T cells (Treg) are a distinct population of suppressive lymphocytes that prevent other immune cells from becoming activated, proliferating, and performing their effector roles. By directing and intensifying both innate and adaptive immunity as well as moderating a variety of host immunological responses, Treg cells are essential for the preservation of host immune homeostasis (Chaudhry and Rudensky, 2013). Treg cells were shown to be enriched in periodontitis lesions in numerous publications on human patients (Nakajima et al., 2005; Cardoso et al., 2008). Interesting results also showed that periodontitis lesions had fewer Foxp3^+^CD25^+^ cells (Ernst et al., 2007). It is still unknown what led to the contradiction. The well-known anti-inflammatory cytokines TGF-β and IL-10 are also important cytokines for Tregs, while their levels of expression were inversely linked with the severity of the periodontitis (Dutzan et al., 2012). In IL-10-deficient animals, *P. gingivalis* infection resulted in more severe alveolar bone loss (Sasaki et al., 2004), and the treatment of IL-10 inhibited alveolar bone loss (Zhang et al., 2014), demonstrating the protective role of IL-10. Research conducted both *in vivo* and *in vitro* demonstrated that *P. gingivalis* can promote the formation of Treg cells (Kobayashi et al., 2011). It’s interesting to note that *P. gingivalis*’s impact on Treg growth depends on the environment. For instance, compared to non-pregnant mice, pregnant mice had less Treg cells stimulated by *P. gingivalis*. Also, the reduction of Tregs in atherosclerotic patients following infection with *P.* gingivalis, particularly in those with type II FimA, suggests that type II FimA may be linked to Treg dysregulation (Yang et al., 2022). As a whole, additional study is needed to comprehend the functions of T-cell subsets in periodontitis and the biological significance of *P. gingivalis*’s modulation of those functions in the context of its function as a keystone pathogen.

## 3 Atherosclerosis

Atherosclerosis is a disorder characterized by inflammation and multifaceted conditions, which is caused by the accumulation of lipid droplets and different types of immune cells in the arterial wall. These cells include macrophages and T and B lymphocytes (Pirro and Mannarino, 2019). Despite being the minority, T and B cells are crucial for the modulation of immune responses during the progression of atherosclerosis (Gounopoulos et al., 2007; Libby, 2012). The subsets of T cells have all been identified in the atherosclerotic plaque of the human body (Andersson et al., 2010; Hansson and Hermansson, 2011; Libby et al., 2011). Additionally, there are growing efforts to find immunomodulatory treatments that target T or B cells with potential antiatherosclerotic effects. Different subtypes of T and B lymphocytes that control various branches of the adaptive immune system have been discovered throughout the progression of atherosclerosis (Pirro and Mannarino, 2019). Although these ideas of immunomodulatory techniques are appealing, clinical translation is greatly hampered by the lack of understanding of the functions of autoantibodies, B and T cells, and the low predictive value of animal models (Libby, 2012).

A 1993 study discovered an association between periodontitis and cardiovascular disease (Mattila et al., 1993; Wolf and Ley, 2019). In 2012, the American Heart Association endorsed these findings and asserted the relationship is unrelated to the earlier established criteria (Mattila et al., 1989). Although the pathogenesis of atherosclerosis is uncertain, *P. gingivalis* is currently gaining attention due to its potential role in accelerating the progression of the disease (Tonetti, 2009; Lockhart et al., 2012). By altering the host’s lipid profile, *P. gingivalis* infections in the mouth have been shown by Maekawa et al. to facilitate the growth of atheroma. Patients with cardiovascular illness were also shown to have high titers of *P. gingivalis* antibodies. (Maekawa et al., 2011). Previous studies have suggested that the invasion of cardiovascular cells and tissues by *P. gingivalis* may contribute to the onset of atherosclerosis (Pussinen et al., 2007; Olsen and Progulske-Fox, 2015).

### 3.1 The discovery of anti-HSP60 antibodies

HSP60 is a chaperonin type, also known as the 60 kDa heat shock protein 60. A chaperonin is a group of proteins that aids in the assembly of intracellular molecules and protein folding. On the cell surface and in the extracellular milieu, HSP60 chaperones also function as danger signals for stressed and injured cells (Pockley and Multhoff, 2008), serving as potent stimulators of the immune responses (Pockley et al., 2008; Cai et al., 2014). Previous research has demonstrated that HSP60 is present in atherosclerotic lesion regions (Kol et al., 1998). High titers of HSP60 antibodies were discovered in individuals with coronary heart disease, carotid atherosclerosis, and stroke. Additionally, a strong association between anti-HSP antibody levels and the prevalence of atherosclerosis was identified. (Mandal et al., 2004). Studies have also revealed that *P. gingivalis* can express GroEL-like proteins from the HSP60 family (Hotokezaka et al., 1994). Furthermore, the immune system may be unable to distinguish between the host and bacterial HSP. Yamazaki et al. discovered that cross-reactive T cells against human HSP60 and *P. gingivalis* HSP60 (GroEL) exist in both periodontitis and atherosclerotic aneurysm tissue (Yamazaki et al., 2004). In addition, Choi et al. discovered that atherosclerosis patients had elevated titers of anti-*P. gingivalis* HSP60 IgG antibody (Choi et al., 2002) and *P. gingivalis* Hsp-specific T-cell lines, which were formed from peripheral blood and atherosclerotic lesions. These data imply that the *P. gingivalis* HSP60-specific T-cell immunological response may be a component of the immunopathologic process underlying atherosclerotic disease.

### 3.2 Th17/Treg imbalance

Atherosclerosis was thought to occur due to a Th1/Th2 imbalance (Cheng et al., 2008). However, recent views have changed due to new evidence that patients with acute coronary syndrome have an imbalance of Th17/Treg (Yang et al., 2017). According to Cai et al., during the development of periodontitis, an infection caused by *P. gingivalis* increases the responses of Th17 cells (Cai et al., 2014). Since a Th17/Treg imbalance causes inflammation, it is possible to propose that plaque destabilization may also be influenced by this imbalance. Moreover, Tregs are known to produce plenty of TGF-β and IL-10, and by repressing immune cell functions, these proteins play a vital role in the pathogenesis of atherosclerosis. The study also revealed that patients with *P. gingivalis* who develop atherosclerosis have lower Treg cell levels than a control group who do not (Yang et al., 2014). A collection of TGF-β1 was reduced in those infected with *P. gingivalis*. According to Cai et al. (2014), *P. gingivalis*-induced atherosclerosis in ApoE-/- mice increased the amount of Th17 cells and Th17-related chemicals in the heart relative to Th1 and Treg cells. The finding may indicate that Th17 cells have potent pro-inflammatory functions to exacerbate the progression of atherosclerosis. Additionally, in *P. gingivalis*-infected ApoE-/- mice, Yang et al. (2017) found that *P. gingivalis* enhances the formation of atherosclerotic lesions and plaque instability. Such a finding was also associated with decreased Treg cell frequency and increased Th17 cell frequency. The evidence suggests that *P. gingivalis* oral infection may contribute to a Th17/Treg imbalance by impacting T-cell differentiation during atherosclerosis, acting as a vital factor in expanding lesion areas and reducing plaque instability.

### 3.3 *P. gingivalis* inhibits IL-2 expression and accumulation

Interleukin-2(IL-2) regulates regulatory T cells and type-2 innate lymphoid cells, the two cells that are crucial for atherosclerosis and myocardial healing (Zhao et al., 2020). Additionally, Khalaf et al. reported that the virulent *P. gingivalis* inhibits the expression and accumulation of IL-2 (Khalaf and Bengtssown, 2012), which results in the production of active oxygen species and the elevation of Ca^2+^ in blood, as well as the depreciation of activity of transcription factors, namely AP-1 and NF-κB. AP-1 has been proven to be an essential regulator of IL-2. The partial suppression of arginine-specific (Rgp) gingipains, a protease secreted by *P. gingivalis*, could also result in the inhibition of IL-2 accumulation. In summary, *P. gingivalis* infection and the inhibition of AP-1 protein both impact the accumulation of IL-2 at the protein level (Khalaf and Bengtssown, 2012). Therefore, it has been proposed that the alteration of adaptive immune response and the fluctuation of IL-2 levels are responsible for the internal inflammatory state of atherosclerosis.

### 3.4 Prevention of atherosclerosis by mucosal vaccination

Avoiding periodontal inflammation may be an effective way to reduce the formation and progression of atherosclerosis since the periodontal infections that cause it may involve various potential mechanisms. Nasal immunization with a 40 kDa outer membrane protein of *P. gingivalis* effectively restrained atherosclerosis and inflammation in C57BL/6 mice fed with ApoEshl and an HFD (Koizumi et al., 2008; Fukasawa et al., 2012). Furthermore, mucosal administration of related autoantigens is an effective way to lessen autoimmune diseases by inducing an unresponsive state of tolerance (Calder et al., 2006) because Hsp60 (GroEL) from *P. gingivalis* can trigger a connection between atherosclerosis and periodontitis (Choi et al., 2004; Choi et al., 2011). Because the acceleration of *P. gingivalis* infection is controlled by *P. gingivalis* GroEL sublingual immunization (Hagiwara et al., 2014), mucosal vaccination with *P. gingivalis* Hsp60 may also be able to regulate the inflammation and the development of atherosclerosis due to the periodontal pathogen infection.

**Figure 1:**
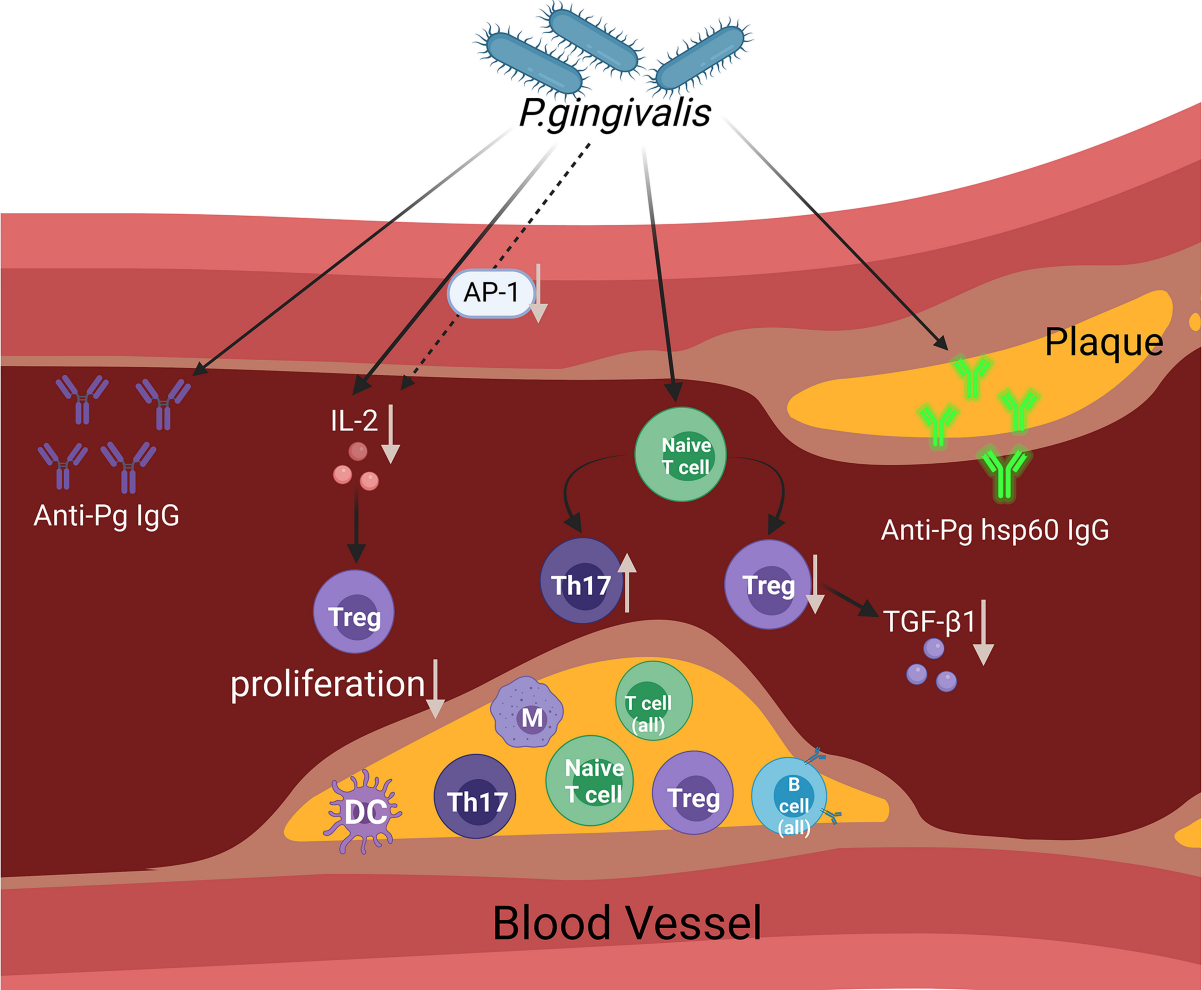
This figure aims to display the presumed contribution of *P. gingivalis* to atherosclerosis *via* the regulation of the adaptive immune system. The figure shows a diagram of an open blood vessel affected by atherosclerotic plaque. Within the diagram, we can see that there are different types of immune cells affected in the blood vessels and atherosclerotic plaques. As infection rises within the blood vessels caused by *P. gingivalis*, the Th17/Treg cell population becomes imbalanced (an increase in the Th17 cell population and a decrease in the Treg cell population) and reduces TGF-β1 levels. In reaction to *P. gingivalis*, AP-1 transcription factor activity was reduced. The accumulation of IL-2 is influenced by *P. gingivalis* and partially affects the suppression of AP-1, which affects Treg proliferation. The involvement of adaptive immune responses of the host is proven by the evidence of increased anti-Pg lgG and anti-Pg hsp60 lgG. (Created with BioRender.com).

The possible mechanisms through which *P. gingivalis* affects the state of arteriosclerosis (mainly adaptive immune response) are summarised in **Figure 1**.

## 4 Hypertension

Hypertension is primarily caused by functional changes in blood vessels, such as increased vasoconstrictor responses and endothelial dysfunction. Recent studies have examined the link between periodontitis and hypertension and successfully linked the two conditions epidemiologically. While there are no simple mechanisms that can fully explain the aetiology behind hypertension, the involvement of both inflammation and immune response has been reported in many studies. *P. gingivalis* infection influences the host’s inflammatory state and immune responses, promoting hypertension, as evidenced by the findings demonstrating the higher risk of hypertension development in patients suffering from periodontitis (Aguilera et al., 2020). Additionally, the outcomes of clinical trials and periodontal intervention have demonstrated a considerable reduction in systolic and diastolic blood pressure after periodontal therapy is provided (Vidal et al., 2013). This outcome offers preliminary evidence that periodontitis affects hypertension.

The findings demonstrate that the immune response is a crucial factor in developing hypertension and related organ injury, with both innate and adaptive immunity associated with hypertension (Harrison et al., 2011). The presence of T cells in the kidneys and vessels of patients suffering from hypertension has also been reported, in addition to the accumulation of circulating T cells that trigger the production of cytokines in Th1 cells, type 1 T helper cells, and Th17 and IL-17 producer cells (Youn et al., 2013; Itani et al., 2016). Although there are ongoing studies on the T-cell activation process and its influence on hypertension and vascular dysfunction, the results are insufficient to fully understand the mechanisms (Meissner et al., 2017). Chronic infections such as periodontitis have also been suggested to alter the population of T-cells. Such a change in the environment primes the host to exhibit heightened immune responses and promotes disorders like hypertension, such as the elevated production of Th1 that leads to hypertension (Shao et al., 2003). In patients suffering from periodontitis, gingival bacterial infection alters the innate and adaptive immune responses, heightening the level of pro-inflammatory cytokines in the systemic circulation. In the event of chronic infection, T cell memories are formed, which can be activated to divide and generate in a heterologous way through the actions of nonspecific antigens and cytokines (Kim et al., 2002; Freeman et al., 2012). The cells can then migrate towards the vasculature and kidneys, altering the function of the vasculature, leading to renal damage and potentially causing hypertension. This discovery provides preliminary evidence for the relationship between periodontitis and hypertension.

A recent animal study supported the hypothesis that high blood pressure is caused by the Th1 immune response triggered by *P. gingivalis* antigens (Czesnikiewicz-Guzik et al., 2019). Studies have also discovered that the activation of systemic T-cells seems characteristic of hypertension and is aggravated by antigen stimulation by *P. gingivalis*. This characteristic later shifted to increased leukocyte infiltration, particularly T-cell and macrophage infiltration, and aortic vascular inflammation. Studies have revealed the elevated expression of the Th1 cytokines IFN-γ, TNF‐α, and TBX21 in the aortas of *P. gingivalis*/IL-12/aluminum oxide-immunized mouse. While the cytokines continued to show elevated expression, IL-4 and TGF‐β expression was invariant. Furthermore, the induced Th1 mice exhibited a more significant rise in blood pressure and endothelial dysfunction in response to a 2-week infusion of a suppressant or a dose of angiotensin II than the control mice. According to the research, Th1 immune responses to bacterial antigens like *P. gingivalis* appear more sensitive to low-dose angiotensin II and other suppressive or pro-hypertensive insults.

## 5 Adverse pregnancy outcomes

Maternal, fetal, and neonatal complications that occur during pregnancy, labor, and postpartum are all classified as adverse pregnancy outcomes (APOs). Modern epidemiologic studies have consistently implied positive connections between periodontitis and APOs (Ide and Papapanou, 2013). Evidence has depicted that *P. gingivalis* DNA and antigens are present within the placenta, umbilical cord (Vanterpool et al., 2016), and amniotic fluid (Leon et al., 2007), which are associated with multiple types of pregnancy complications (Chaparro et al., 2013). These researches suggest that *P. gingivalis* directly invades and damages uterine placental tissues, contributing to the development of APOs. A study confirmed that *P. gingivalis* infection could increase the risk of uterine-placental pathologies, including endometrial arteritis, mild chorioamnionitis, and uterine-placental hemorrhage accompanied by placental structural disorders. Thus, we further sought to prove that *P. gingivalis* infection contributes to the development of APOs.

### 5.1 Th1/Th2 cytokine increase in ratio

It is known that periodontitis causes immune responses to switch from Th2 to Th1 and releases cytokines that stimulate the Th1 immune response (Raghupathy, 2001). Fetal growth restriction, spontaneous abortion, and maternal rejection of the implanted fetus have been linked with an increase in the expression of Th1-mediated cytokines such as TNF-α, IL-1, IL-8, IL-2, IL-12, and IFN-γ, as well as a decrease in the expression of Th2-mediated cytokines such as IL-10, TGF-β2, and IL-6 (Liang et al., 2006).

### 5.2 Th17/Treg imbalance and the increased production of IL-17

Multiple studies have proposed that an imbalanced Th17 profile (surplus) and Treg cells (reduced) may lead to the development of APOs (Figueiredo and Schumacher, 2016). Pregnancy-induced hypertension, fetal growth restriction, and recurrent miscarriage are the pregnancy-related complications associated with such imbalance. These pregnancy-related complications and maternal inflammation have been associated with the increasing of Th17-mediated cytokines (Dechend et al., 2000; Pongcharoen et al., 2006; Keeler et al., 2009; Dhillion et al., 2012; Ozkan et al., 2015). Additionally, the production of Th17-related cytokines, such as IL-17, was reportedly increased following the activation of Th17 cells by *P. gingivalis*. According to the main genotype subunit of fimbriae FimA, the strains of *P. gingivalis* can be classified into six types: I, Ib, II, III, IV, and V. The W83 strain, a type IV strain, is classified as a highly virulent strain of *P. gingivalis*, which is also a potent inducer of Th17-mediated IL-17 production. A study demonstrated that the activation of NF-κb and RORγ by *P. gingivalis* caused the elevated production of IL-17 (Manel et al., 2008). Furthermore, *P. gingivalis* can selectively suppress IL-12 an and boost the Th17 lineage by varying the Th17 to Treg cell ratio (Moutsopoulos et al., 2012), a mechanism associated with the pathogenesis of preeclampsia and spontaneous abortion (Keeler et al., 2009). Additionally, Pngcharoen et al. discovered a significant increase in progesterone secretion during labor due to Th17-induced IL-17 (Pongcharoen et al., 2006).

IL-17 regulates the remodeling of the uterine spiral artery and the modification of the superficial invasion of EVTS into uterine tissues. During the pregnancy period, the progression of atherosclerosis, FGR, and preeclampsia are believed to be associated with the remodeling of the altered spiral artery (Keeler et al., 2009; Ozkan et al., 2015). Ozkan et al. discovered that compared with the control groups, patients suffering recurrent/idiopathic miscarriages and patients with preeclampsia exhibited relatively high levels of IL-17, IL-23, IL-6, soluble IL-6 receptor, and RORγ (Park et al., 2012b; Ozkan et al., 2015). In addition, the production of IL-17 in fetal and placental tissues was increased due to the regulation of angiotensin II type I receptor (AT1-AA) and increased placental oxidative stress caused by *P. gingivalis* (Dechend et al., 2000). Furthermore, IL-17 production enhanced by AT1-AA has also been linked with the progression of atypical cytotrophoblast invasion and the remodeling of inferior spiral arteries. Recently, a correlation between the increased expression of IL-17 and IL-17R and abnormal cortical progression and autism-like behavioral abnormalities in the developing embryo has been revealed (Dechend et al., 2000; Dhillion et al., 2012).

## 6 Inflammatory bowel disease

Inflammatory bowel disease (IBD) is a class of chronic idiopathic diseases, primarily composed of: Crohn’s disease (CD) and ulcerative colitis (UC). Today, multiple epidemiological studies have suggested a link between IBD and periodontitis. Compared with non-IBD patients, patients suffering from IBD have been found to have a relatively high risk of periodontitis and weak oral health (Papageorgiou et al., 2017; Lauritano et al., 2019). A study on SAMP1/YitFc mice revealed a spontaneous model of Crohn’s disease generated by natural periodontitis (Pietropaoli et al., 2014). Additonally, IBD patients’ oral symptoms also have an impact on the composition of the oral microbiota (Xun et al., 2018), indicating a connection between IBD and PD.

Despite the findings, the pathogenesis of inflammatory bowel disease is still poorly understood, which may be related to environmental factors and genetic elements, intestinal disorders, and immune regulation disorders (de Souza and Fiocchi, 2016). Furthermore, the combination of a genetic predisposition, an excessive host response, and the presence of environmental stimuli, which are the primary factors in the pathogenesis of periodontitis, are comparable to those that cause IBD (mainly pathogenic microflora). Multiple factors affecting IBD have also been identified as risk factors for periodontitis (D'Aiuto et al., 2004; Kinane and Bartold, 2007; Indriolo et al., 2011). Additionally, studies have proven that both IBD and periodontitis diseases exhibit the characteristics of immunoinflammatory and tissue destruction (Brandtzaeg, 2001; Kinane and Bartold, 2007). The underlying mechanisms that connect these two diseases may include common bacterial aetiology and shared immune pathways (Baima et al., 2022). First, intestinal ecology becomes dysbiotic due to the migration of periodontal pathobionts from the mouth cavity to the gut. Second, a disturbed gut microbiota results in an intestinal immune response that manifests as intestinal and systemic inflammation, which can cause periodontitis to develop or worsen. And last, the original causes of PD or IBD can be dysbiosis of the intestinal or oral microbiota. Subsequent modifications, such as pathogens, virulence factors, toxic metabolites, and other proinflammatory elements, might travel *via* the circulatory system between the intestines and the mouth cavity. Also, Stein et al. conducted a study to examine periodontal pathogens in the subgingival plaque of 147 Crohn’s disease patients (Stein et al., 2010), which revealed that among all 147 patients, 76.9% were found infected by *Aggregatibacter actinomycetemcomitans* and 62.6% were found infected by *P. gingivalis*. Combined with the recent animal studies showing that periodontal pathogens can cause intestinal inflammation, it is safe to conclude that there is a bidirectional relationship between the two diseases.

A survey by Arimatsu et al. depicted that the oral administration of *P. gingivalis* changed the C57BL/6N mouse gut microbiota (Arimatsu et al., 2014), in addition to the change in the function of the gut epithelial barrier, increasing gut permeability. *P. gingivalis* infection also disrupted the colonic epithelial barrier due to decreased tight junction protein expression (Tsuzuno et al., 2021). In addition to the impairment in intestinal barrier function, the administration of *P. gingivalis* also induced the overexpression of pro-inflammatory cytokine mRNA in the intestine. The mRNA expression of IL-6 and TNF-α was also observed in the small and large intestines. An insignificant increase in the expression of Foxp3, a characteristic regulatory T cell marker, was also reported, in addition to the reduced expression of Rorγt, a distinct Th17 cell marker (Nakajima et al., 2015). Additionally, in the DSS-induced colitis model, it was discovered that the intravenous injection of *P. gingivalis* extract-stimulated CD4+ T cells aggravated the inflammatory response and increased the Th17/Treg ratio in the colon and lamina propria lymphocytes. And CD4+ T cells stimulated with *P. gingivalis* and *Lactobacillus rhamnosus GG* were found to alleviate colitis by reducing the Th17/Treg ration *via* JAK-STAT signaling pathway (Jia et al., 2020). Zhao et al. found that *P. gingivalis* exacerbated the severity of UC, in part through *Porphyromonas gingivalis* peptidylarginine deiminase (PPAD). The main mechanisms are stimulation of Th17 numbers and IL-17 production, and reduction of Treg numbers and IL-10 production (Zhao et al., 2021). According to a recent study, *P. gingivalis* modifies the gut microbiota, which eventually stimulates intestinal IL-9+CD4+T cells and inflammation (Sohn et al., 2022). The finding implies that the rise in regulatory T cells may counteract the rise in intestinal tissue inflammation brought on by *P. gingivalis*, but more research in this area is still needed.

## 7 Diabetes mellitus

Diabetes mellitus (DM) is a category of metabolic illnesses distinguished by persistent hyperglycemia that develops as a result of irregular insulin production and/or insulin resistance over an extended period of time. Type 1 diabetes mellitus (T1DM) and type 2 diabetes mellitus (T2DM) are the two most common kinds of DM. T1DM results from autoimmune destruction of β-cells, which typically causes an utter lack of insulin and latent autoimmune diabetes of maturity. T2DM results from insulin resistance and a progressive loss of sufficient β-cell insulin production (American Diabetes Association, 2021). Diabetes mellitus raises the risk of periodontitis, despite the absence of any phenotypic characteristics specific to periodontitis in patients with the condition. Periodontitis also has an impact on the complications of glycemic control. Due to the “two-way” nature of the association between periodontitis and diabetes mellitus, numerous clinical and experimental research have been conducted to better understand the molecular mechanisms underlying these two diseases and how they might interact. Here, we mainly reviewed the important role played by *P. gingivalis* in the bidirectional relationship between periodontitis and diabetes.

The advancement of next-generation sequencing technologies has aided in the research of the human microbiome, especially the oral microbiome and associated systemic disorders, in recent years. Recent studies have shown that people with diabetes mellitus are at risk for developing periodontitis because of a decline in the relative abundance and prevalence of species compatible with health and an increase in the pathogenic content of the periodontitis-associated microorganisms (including *Porphyromonas, Prevotella, Campylobacter*, and *Fusobacterium*) (Ganesan et al., 2017; Long et al., 2017). Some authors, however, present findings that type 2 diabetes mellitus decreases reduces the variety and density of the subgingival microbiome, that this decline is even connected to insufficient glycemic management (Longo et al., 2018; Saeb et al., 2019). Another glycemic control study of the periodontal microbiota showed that in patients with moderate or severe periodontitis, the orange-red cluster *(Prevotella melaningenica, Prevotella intermedia, Prevotella nigrescen*, and *P. gingivalis* mixture) was inversely associated with fasting glucose levels (Merchant et al., 2014). This study has demonstrated a relationship between periodontal antibody titers and hyperglycemia. Despite being highly relevant, there are still a lot of uncertainties about the makeup of the subgingival microbial community in diabetes circumstances.

Increased inflammation is typically associated with diabetic problems, and there is strong evidence that DM enhances periodontal tissue inflammation. In human periodontal tissues, T1DM and T2DM both cause an increase in the release of inflammatory cytokines and chemokines (Bastos et al., 2012; Polak and Shapira, 2018). These cytokines include IL-1β, IL-17, IL-23, IL-6, and tumour necrosis factor (Salvi et al., 1998; Graves et al., 2020). Contrarily, diabetics have lower levels of anti-inflammatory factors including IL-4, IL-10, and TGF-β, along with M2 macrophages and anti-inflammatory regulatory T cells (Acharya et al., 2017; Van Dyke, 2017).

The effects of periodontitis on diabetes mellitus may generally be correlated with bacteria and the incidence of inflammatory cytokines in systemic circulation. Therefore, a heightened systemic inflammatory response to subgingival bacteria results in systemically elevated levels of pro-inflammatory cytokines that promote insulin resistance. An interesting experimental study of periodontitis in C57BL/6 female mice induced by various oral pathogens *(P. gingivalis, F. nucleatum, P. intermedia*) depicted that pathogen-induced periodontitis elevated insulin resistance in high-fat diet-fed mice. Such an event is believed to be caused by the adaptive immune system’s response that explicitly targets periodontal disorders-causing pathogens. Empirical evidence also demonstrates that periodontitis simultaneously affects local and systemic immune responses, weakening glucose metabolism. Moreover, it was found that the periodontitis-aggravated metabolic disease is protected through the transfer of cervical lymph node cells from the infected mice to the noninfected recipients. Treatment with inactivated *P. gingivalis* before the periodontal infection resulted in the generation of specific antibodies against *P. gingivalis*, which protects the mouse from periodontitis-induced dysmetabolism. The findings prove the relationship between *P. gingivalis* antibodies caused by periodontitis, the decrease in such specific antibodies, and defective glucose metabolism in HFD-fed mice. This leads to the conclusion that regional (cervical) adaptive immune system modulation is a partial cause of insulin resistance induced by periodontitis (Blasco-Baque et al., 2017). The findings suggest that vaccination against *P. gingivalis* may lessen the effect of periodontitis on glucose metabolism. However, the role of *P. gingivalis* monomers in the two-way relationship between periodontitis and diabetes requires a lot of in-depth research.

## 8 Non-alcoholic fatty liver disease

Today, it is acknowledged that non-alcoholic fatty liver disease (NAFLD) is one of the most prevalent chronic liver illnesses in people who consume little or no alcohol, also having a fatty liver (Chalasani et al., 2018). NAFLD is an overall term for non-alcoholic steatohepatitis (NASH), a risk factor for fibrosis and liver cancer, and simple fatty liver with little or no inflammation. Numerous studies on NAFLD have been conducted, including both cross-sectional and prospective epidemiological studies, which have proven that periodontal disease is a risk factor for NAFLD (Alazawi et al., 2017; Iwasaki et al., 2018; Shin, 2020). Evidence from the *in vivo* study of animal models demonstrates that the development of NAFLD is accelerated, and steatosis is strengthened by the periodontopathic bacterial infection (Yoneda et al., 2012; Furusho et al., 2013; Komazaki et al., 2017). In addition, the presence of periodontopathic bacteria was identified in the liver, further suggesting the direct impact of *P. gingivalis* on NAFLD development. Another study showed significantly higher levels of *P. gingivalis* in patients with NAFLD than in those without NAFLD (Yoneda et al., 2012).

### 8.1 Increasing anti-*P. gingivalis* titers and *P. gingivalis* in patients with NAFLD

Fim A type 2 *P. gingivalis*, which exhibits significant adherence and invasion to host cells and is linked to severe periodontitis (Amano et al., 1999; Nakagawa et al., 2002), was found half of the individuals with NAFLD (Yoneda et al., 2012). Previously, 200 individuals with NAFLD had their livers biopsied and their serum anti-*P. gingivalis* antibody titers were measured. The degree of fibrosis and antibody titers against *P. gingivalis* fim A types 1 and 4 were found to be positively correlated. Additionally, progressive fibrosis was linked to antibody titers against *P. gingivalis* fim A type 4. (Nakahara et al., 2018). Immunohistochemistry was used to identify *P. gingivalis* in the hepatocytes of liver biopsy samples taken from 40 NAFLD patients. According to the findings, those who tested positive for *P. gingivalis* progressed more quickly toward hepatic fibrosis than those who tested negative (Furusho et al., 2013). In addition, many studies have shown that *P. gingivalis* lipopolysaccharide can induce hepatocyte inflammation and intracellular lipid formation (Furusho et al., 2013; Zaitsu et al., 2016; Ding et al., 2019).

### 8.2 The imbalance of Th17/Treg

However, findings of *P. gingivalis* affecting the mechanism of adaptive immunity in NAFLD remain limited. Recent studies have revealed that Th17 cells are abundant in animal models’ liver and peripheral blood with NAFLD (Fabbrini et al., 2013; Guo et al., 2016; Rolla et al., 2016). The Th17 cell count in periodontal disease tissues was reportedly high due to *P. gingivalis* infection. Therefore, when examining the connection from an immunological perspective, *P. gingivalis* infection may activate the Th17 axis *in vivo*, and Th17 cells produced by periodontitis may move to the liver and worsen NAFLD (Hatasa et al., 2021). A recent study also found that oral *P. gingivalis* treatment can directly induce NAFLD in mice, which may be reliant on ferroptosis of liver cells that took place through the imbalance of Th17/Treg brought on by disrupted microbial metabolism (Yao et al., 2022). Meanwhile, insulin resistance is a significant pathogenic factor for NAFLD. Mice suffering from endotoxaemia caused by the injection of *P. gingivalis* exhibited enhanced NAFLD and damaged glucose tolerance and insulin resistance (Sasaki et al., 2018). The findings demonstrate that the *P. gingivalis* mechanism related to insulin resistance discussed above may also impact NAFLD, although more research is needed to identify associated processes.

## 9 Rheumatoid arthritis

Rheumatoid arthritis (RA) is a chronic inflammatory autoimmune disease that damages the tissues in the joints, sometimes resulting in functional impairment (Scott et al., 2010). Recently, several epidemiological studies have found an association between periodontal disease and RA, suggesting that individuals with periodontal disease have a higher prevalence of RA than healthy controls (Ranade and Doiphode, 2012; Fuggle et al., 2016). These findings encourage more research into the similarities between pathogenic and clinical traits of these disorders (Potempa et al., 2017). Patients were found to have autoantibodies, including anti-citrullinated peptide antibodies (ACPAs), years before the disease even manifests itself, suggesting that the immune reactions toward the condition are triggered at sites other than the joints (Rantapaa-Dahlqvist et al., 2003; van de Sande et al., 2011). The development of the immune response in RA is believed to originate from the infection by lung microbiota, periodontal disease, and oral microbiota.

**Figure 2:**
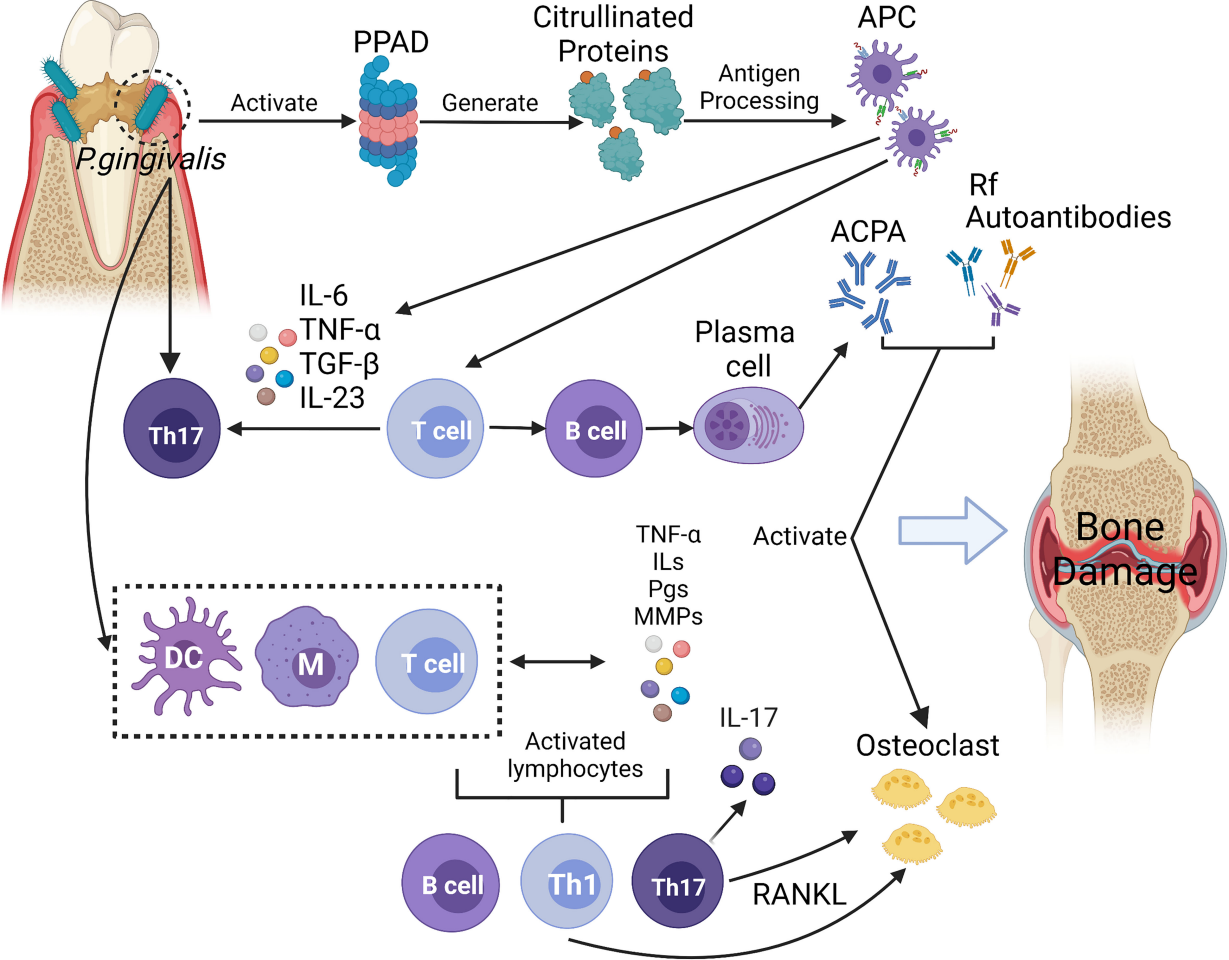
In this diagram, *P. gingivalis* infection displays a series of activities and effects that lead to bone and cartilage damage. The infection activates proteases and peptidylarginine deiminase (PPADs), which generates citrullinated proteins and triggers the synthesis of anti-citrullinated protein antibodies (ACPAs). In the above reaction, antigen presenting cells (APC) can not only activate T cells and promote B cells’ autoantibodies production, but also produce inflammatory cytokines (such as IL-6, IL-23, IL-1β) to induce Th17 cells, which also affect the development of RA. With the combination of the inflammatory process stimulated by macrophages, dendritic cells (DCs), and T cells, the host response towards citrullinated proteins occurs. With this response, immune cells begin to produce proinflammatory mediators such as interleukins (ILs), prostaglandins (PGs), tumour necrosis factor (TNF), and metalloproteinases (MMPs). The mediators mentioned above also contribute to the irritation of immune responses. IL-17, an important cytokine of Th17 cells, induces osteoblast expression of receptor activator of the factor nuclear kappa B ligand (RANKL), which stimulates osteoclast activation. Enhanced expression of rheumatoid factor (RF) and ACPAs is caused by a resultant signal against citrullinated epitopes in the joints, and such enhanced expression then aids in the formation of immune complexes. These immune complexes form to empower the host’s inflammatory development, which may further complicate RA. Furthermore, ACPAs, RF and other autoantibodies (such as antibodies against carbamylated proteins (anti-CarP), anti-acetylated protein antibodies (AAPA)) may also contribute to the inflammatory process by activating osteoclasts directly and resulting in bone and cartilage damage. (Created with BioRender.com).

Due to their numerous similarities in pathological and immunological characteristics, the link between periodontitis and RA has been noted. First, *Porphyromonas gingivalis* peptidylarginine deiminase (PPAD) was directly related to the formation of ACPA. Moreover, the production of pro-inflammatory mediators and the increased infiltration of immune cells induced Th17 cell response, which affect the development of RA. In addition, immune cells were induced to release receptor activator for nuclear factor-κB ligand (RANK-L) that resulted in activating osteoclasts (de Pablo et al., 2009). The possible pathogenic mechanisms linking *P. gingivalis* to RA are briefly summarised in **Figure 2**.

### 9.1 PPAD catalyzeds protein’s citrullination and lead to the production of ACPA

*P. gingivalis* is thought to be the causative agent of the reactions described above because of its unique ability to citrullinate proteins *via* endogenous *Porphyromonas gingivalis* peptidylarginine deiminase (PPAD) (McGraw et al., 1999a; Rodríguez et al., 2009). The highly conserved PPAD gene is widespread within *P. gingivalis* but is absent from *P. gingivalis*-related species. Lappin et al. discovered that the levels of ACPA serum in patients suffering from periodontitis induced by *P. gingivalis* were higher than in patients without *P. gingivalis* (Lappin et al., 2013). It was also found that despite the distribution of *P. gingivalis*, antibody responses against *P. gingivalis* were more potent in patients diagnosed with RA and severe periodontitis than in patients in the control group who suffered only from the periodontal condition (de Smit et al., 2012). In contrast to human PAD, PPAD can be activated at higher pH levels and largely functions by citrullinating C-terminal arginines. Citrullinated peptides are produced when arginine gingipains (Rgp) collaborate with another enzyme to degrade polypetides into short peptides with C-terminal arginines, which is subsequently followed by a speedy citrullination process assisted by PPAD (McGraw et al., 1999a; Rodríguez et al., 2009). The continuous exposure to citrullinated peptides that are localized at the level of the periodontium may be caused by P. gingivalis’ posttranslational changes, which may cause immunological tolerance in people with certain genetic dispositions to break down. Lastly, this might influence the creation of ACPA (Valesini et al., 2015). After the infection of *P. gingivalis*, neutrophils have the ability to extract neutrophil extracellular traps, which are structures with active proteases and PADs that produce citrullinated epitopes and cause ACPA to be produced. Additionally, neutrophils may be drawn to a gingival fissure that is undergoing necrosis, releasing molecules that are associated with damage and causing both local and systemic inflammation (Hahn et al., 2013; Khandpur et al., 2013; Pratesi et al., 2014). Collagen-induced arthritis (CIA) is the most universally studied model of rheumatoid arthritis. A model was created using an inflammation-prone mouse strain by inoculating the mouse with *Aggregatibacter*, *Actinomycetemcomitans*, and *P. gingivalis* orally to induce arthritis and experimental periodontitis. In the study, neither periodontitis nor arthritis progression was altered in the control mice carrying pristine-induced arthritis. The findings reveal that the exacerbation of CIA in DBA/1 mice seems to be dependent on the expression of PPAD (Maresz et al., 2013), demonstrating the crucial role of PPAD in the relationship between periodontitis and RA. Gully et al. found that in the BALB/c mice orally inoculated with a PPAD knockout *P. gingivalis* W50 strain; the CIA intensity was lower than in the *P. gingivalis* W50 wild-type strain (Gully et al., 2014). In addition, mice infected with PPAD-KO *P. gingivalis* exhibited a lower level of ACPA serum than the wild-type strain. Multiple studies have suggested PPAD as a critical factor linking the pathogenic hypothesis between RA and periodontal disease.

### 9.2 *P. gingivalis* stimulates the production of pro-inflammatory mediators and induces Th17 response

*P. gingivalis* can also stimulate immune cells to produce pro-inflammatory mediators, such as IL-6, IL-1β, TNF-α, IL-17, and matrix-degrading enzymes (MMPs) (Gonzales et al., 2014). These overproduced pro-inflammatory mediators were also the critical pathogenesis of RA, which led to the cartilage and bone destruction (McInnes and Schett, 2011). In addition, through a model of T-cell-dependent experimental arthritis, evidence of the relationship between periodontitis and RA *via* cellular immunity was discovered. In a study on DBA/1 mice, the severity of CIA was significantly enhanced by periodontitis induced by *P. gingivalis* and *Prevotella nigrescens*, as characterized by the increase in arthritic bone erosion *via* an antigen-induced Th17 response (de Aquino et al., 2014). Th17-mediated immunity was observed to intensify the inflammatory responses to both periodontitis and RA in mice carrying both conditions, and it was reliant on a common hyperinflammatory genotype (Trombone et al., 2010; de Aquino et al., 2014). Thus, the bond between RA and periodontitis may be linked to the mutual aggravation of inflammatory and immunity responses (de Smit et al., 2012). Th17-mediated immunity has gained more attention due to research on the relationships between human periodontitis and RA (Leipe et al., 2010; Lin et al., 2017).

### 9.3 *P. gingivalis* induces the production of RANK-L and promotes the development of osteoclastogenesis

Other virulence components, such as lipopolysaccharide, gingipains, lipoproteins, and fimbriae, are also produced by *P. gingivalis*. Receptors such as protease-activated receptors, Toll-like receptors, and NOD2 receptors recognize these virulence components in gingival epithelial cells and phagocytes, leading to the activation of RANK-L signaling pathways, complement system, and helper T cell differentiation, which eventually contribute to the development of osteoclastogenesis (Lu et al., 2018; Narayan et al., 2018). Persistent *P. gingivalis* infection induced the increase of pro-inflammatory mediators and chemokines such as TNF-α, IL-17, IL-6, IL-1β, RANK-L and so on (de Pablo et al., 2009). RANK-L was the main regulator of osteoclastogenesis (McInnes and Schett, 2007; McInnes and Schett, 2011). In addition, activated lymphocytes (especially Th1 and Th17 cells) further stimulated the production of RANK-L (de Pablo et al., 2009), ultimately exacerbating inflammation and osteoarticular destruction in RA.

## 10 Alzheimer’s disease

Alzheimer’s disease (AD), a progressive neurological disease, is a common type of dementia, accounting for between 60 and 80 percent of all cases (Alzheimer’s Association, 2016). AD is characterized by disrupted cognition, classically amyloid-beta (Aβ) deposits, and hyperphosphorylated neurofibrillary tangles (Goedert et al., 1991). Numerous clinical and experimental research have suggested that systemic peripheral inflammation or infection may be the crucial factor in the pathophysiology of AD, which supports bidirectional communication between the brain and the systemic immune response (Perry et al., 2007; Poole et al., 2013; Cunningham and Hennessy, 2015).

### 10.1 An increasing of Aβ deposition

Early research on Alzheimer’s disease has typically assumed that innate immunity is the primary factor contributing to neuropatholog. Recent studies have revealed a different perspective, suggesting that the adaptive immune system influences the mechanisms related to suppressing neuropathology in AD (Marsh et al., 2016; Olsen et al., 2016). Although the innate immune system is thought to be linked to inflammatory components in AD, adaptive immunity was not previously thought of. Additionally, the role of adaptive immunity has been proposed based on genome-wide studies, which reveal the association between the inability of microglia to clear Aβ from the brain and several genes involved in the immune system (Guerreiro et al., 2013). Pathogens can typically increase Aβ deposition, including *P. gingivalis* lipopolysaccharide (Wu et al., 2017). Aβ is a protein with various antimicrobial effects against bacteria, fungi, and viruses, and its deposition has been linked to the immune response in the host towards the cerebral invasion of pathogens, which are associated with AD (Soscia et al., 2010; Bourgade et al., 2015; Kumar et al., 2016). The peripheral pools of Aβ in the body can be increased by systemic infection with *P. gingivalis* (Nie et al., 2019), and chronic *P. gingivalis* infection may interact synergistically with cerebral RAGE expression and peripheral Aβ production (Zeng et al., 2021). In mice study, an oral *P. gingivalis* infection led to brain colonization and increased production of Aβ_1-42_, a substance found in amyloid plaques (Dominy et al., 2019a).

### 10.2 *P. gingivalis* and virulence factors translocate into the brain

More clinical research has now examined the link between periodontitis and AD, yielding better findings (Stein et al., 2012; Kamer et al., 2015; Chen et al., 2017). A recent research used an animal model of periodontitis and human brain tissues suffering from AD after death. According to the study, *P. gingivalis* and gingipain were translocated into the brain (Ilievski et al., 2018; Dominy et al., 2019a). After receiving oral *P. gingivalis* inoculations every two days for five months, Ilievski et al. found that C57BL/6 mice developed neuropathology similar to Alzheimer’s disease (Ilievski et al., 2018). Dominy et al. also reported evidence showing the influence of *P. gingivalis* and gingipains on the pathogenesis of AD. The study also provided strong evidence proving the presence of *P. gingivalis* DNA and gingipain antigens in the host brain of AD patients (Dominy et al., 2019a). Furthermore, the same study also discovered that by providing small-molecule gingipain inhibitors or the wide-range antibiotic moxifloxacin, the neurodegeneration caused by gingipain could be blocked. Doing so significantly decreased the *P. gingivalis* load in the aged mouse brain and reduced the host Aβ_1–42_ response towards *P. gingivalis* brain infection. This discovery has created a new framework for treating AD (Dominy et al., 2019a).

### 10.3 The increasing of BBB permeability causes an influx of peripheral immune cells and pathogens

As more findings from the studies are revealed, it is possible to suggest that periodontitis-related low-grade systemic inflammation could increase the blood-brain barrier’s permeability (BBB). The increase in BBB permeability causes an surge of peripheral immune cells and pathogens toward the host, which can lead to activate the neuroinflammatory reaction result in the neural network breakdown (Sadrameli et al., 2020). The ability of pathogens to evade the peripheral compartment has further been proven by the discovery of LPS in the neocortex of AD patients (Zhao et al., 2017). Additionally, Poole et al. discovered the presence of *P. gingivalis* lipopolysaccharide in the brains of patients suffering from AD (Poole et al., 2013). Moreover, TLR-2 was found to mediate IL-10 production, which inhibited the IFN-γ and T cell response after the initial systemic exposure to *P. gingivalis*, allowing the pathogen to evade the host’s immune response in the brain (Poole et al., 2013). Through studies conducted in mice, IFN-γ has been found to regulate the protection and repair of neurons. Increased neurogenesis could be an indication of how the immune system normally regulates inflammation and healing in the brain (Baron et al., 2008; Hohlfeld, 2008). Given the evidence, the systemic and oral connections between periodontitis and AD are impossible to ignore. More in-depth studies are required to understand the interactions between the host and pathogen throughout AD development.

## 11 Conclusions and perspective

In summary, many epidemiological studies, *in vivo* experiments, and *in vitro* experiments have demonstrated multiple correlations between periodontitis and many systematic chronic diseases. *P. gingivalis* can affect the adaptive immune system in various ways in periodontitis and many other related systemic disorders. Additionally, strategies that promote immune-subverting and pro-inflammatory mechanisms in periodontal bacteria will harm the periodontium and affect the association of periodontitis with systemic diseases. However, current studies on *P. gingivalis* in affecting systemic diseases through regulating adaptive immunity are insufficient, particularly on neurological disorders, respiratory diseases, and kidney diseases, which are closely related to periodontitis.

Key findings from these studies must be applied in clinical applications, e.g., through the host modulation therapies that combat the disruptive immune mechanisms of periodontal bacteria, thereby assisting in the management of periodontitis and related systemic inflammatory diseases. The host modification strategies may be more effective than direct antibacterial treatments. It is especially crucial when targeting keystone pathogens as they are highly virulent even in low abundance and have a lower chance of being entirely eradicated, partly due to their ability to conceal themselves within permissive host cells. Immunotherapy may be a viable new treatment for chronic inflammatory diseases, which demands further research into the field of oral microbiome modulation and its implications on systemic disease.

## Author contributions

CL and YD participated in the review selection and design. CL and RY wrote the first draft of the review. YD edited and finalized the manuscript. All authors contributed to the article and approved the submitted version.

## Funding

This work was supported by the National Natural Science Foundation of China(No.81800984), Fundamental Research Funds for the Central Universities(No.2020kfyXGYJ082), National Science Foundation of Hubei Province(No.2020CFB787). Free Innovation pre-Research Foundation of Wuhan Union Hospital(2021xhyn090).

## Conflict of interest

The authors declare that the research was conducted in the absence of any commercial or financial relationships that could be construed as a potential conflict of interest.

## Publisher’s note

All claims expressed in this article are solely those of the authors and do not necessarily represent those of their affiliated organizations, or those of the publisher, the editors and the reviewers. Any product that may be evaluated in this article, or claim that may be made by its manufacturer, is not guaranteed or endorsed by the publisher.
